# Book Reviews

**Published:** 1917-10

**Authors:** 


### BOOK REVIEWS

**Books mentioned may be inspected at and ordered through this office. So far as possible, books received in any month will be reviewed in the issue of the second month following. Pamphlets, quarterly and similar periodicals, reports, transactions, etc., will, as a rule, merely be mentioned.**

**Birth Statistics, Registration Area of U. S., 1915.** Bureau of the Census.

776,304 live births occurred—24.9: 1,000 population. The average death rate was 14: 1,000. The birth rates ranged from 21.1 for Me. to 26.7 for Conn. and Mich., the death rates from 10.1 for Minn. to 16.1 for N. H. If there were neither immigration nor emigration, the increase of population would be 1.1% a year or (calculated as compound interest) 11.08 +% for a decade, as compared with an actual increase of 20 down to about 18% for the last few decades. But, obviously, if the population increased only by births, the introduction of a highly vulnerable element would result in a considerably higher mortality. The mortality for infants under one year averaged 10%, ranging by states from 7 for Minn. to 12 for R. I. and, by municipalities of 10,000-25,0000, from 17 for Newburyport, Mass. to 375 for Lackawanna, N. Y. The sanitary and social conditions of Lackawanna are conspicuously bad but, even so, the fact that this city has the maximum infant mortality for the whole country, is due to the large number of inmates of the infant asylum where a relatively high death rate is inevitable, in spite of the best care.

**Therapeutics, Materia Medica and Pharmacy.** Samuel O. L. Potter, A. M., M. D., M. R. C. P., 1st Major and Surgeon of Volunteers, U. S. Army. 13th edition by Elmer H. Funk, M. D., Philadelphia. R. Blakiston's Son & Co., Philadelphia; 960 pages, $6.

The usual introductory matter, definitions, description of Galenicals, constituents of organic drugs, dosage, etc., occupies 50 pages. Materia Medica and Therapeutics, following an alphabetic arrangement, occupy nearly 450 pages; Pharmacy and Prescription Writing, over 50; Special Therapeutics, including an alphabetic index of indications arranged by disease names, poisoning, temperature, clinical examination of the urine, over 300 pages. The Appendix occupies over 100 pages and covers prescription Latin, tables of differential diagnosis, metric and other tables of weights and measures, and the Federal Narcotic Law. A general index follows. Neither space nor words are wasted and, by the classification and index, the division of the book by thumb indexes and use of black letters, rapidity of reference is insured.

---

**Alcohol.** Its Relation to Human Efficiency and Longevity. Eugene Lyman Fisk, Medical Director, Life Extension Institute, authorized by same, published by Funk & Wagnalls Co., N. Y. 216 pages, $1.

We congratulate the author on having dealt with a trite subject, in the orthodox way, in an extremely interesting manner and in really finding something new to say.

---

**Popular Manual of Vocal Physiology and Visible Speech.** Alexander Melville Bell, published by the Volta Bureau, Washington. 3d edition.

The author starts with the actual possible positions of the speech organs, tongue, palate, lips, etc., and, somewhat after the method apparently adopted in forming the Hebrew alphabet, visualizes them by conventional signs which are really diagrams of the actual positions. Thus, related sounds such as B, P, V, F, and M are represented by "letters" of the same basic type, modified to distinguish between sonant and surd mutes and fricatives. The same modifying marks are used in the same way for the D and G groups of sounds. The general plan covers both vowels and consonants, though the type of "letters" for these two fundamental divisions of elementary speech sounds is equally marked. The great advantage of this method is that it not only clearly shows the physiologic act required to produce an elementary sound but that it is elastic enough to cover any possible variation of sounds according to dialectic peculiarities and to denote any sound occurring in any language. Theoretically, at least, an entirely unfamiliar sound—for example the sonants corresponding to the two sounds of ch in German, which do not occur in any language with which the writer is familiar but which are occasionally heard in grunts of disgust—can be accurately transcribed and its pronunciation indicated. One point should be emphasized for authors of future general physiologies: this author clearly recognizes that vowels like consonants are produced in the oral cavity, not in the larynx, as has been falsely taught. Like every subject based on the interpretation of sensory stimuli, there is room for difference of opinion. For example, Wh, seems to the reviewer as clearly a consonantal diphthong, though the letters are reversed. The relation of W and Y to H is another point of divergence of opinion and others might be mentioned in regard to vowels and especially diphthongs. However, the author has devised a thoroughly rational system of transcribing speech and any dispute as to facts should be settled by phonographic study, including the appearance of tracings. Whatever slight modifications are required either by revision of the interpretation of sounds or for practical use in writing and printing will not disturb the foundations of the system.

---

**The Practical Medicine Series,** Chas. L. Mix, A. M., M. D., Editor; The Year Book Publishers, Chicago. Vol. 4, Gynaecology, Emilius C. Dudley, A. M., M. D. and Sydney S. Schochet, M. D., Editors, 232 pages, $1.35 separately. Vol. 5, Paediatrics, Isaac A. Abt, M. D. and A. Kevinson, M. D., Editors; Orthopaedic Surgery, John Ridlon, A. M., M. D. and Chas. A. Parker, M. D., Editors, 240 pages, $1.35 separately. Price for the annual series of 10 volumes, $10.

This series is a standard review of current literature, too well known to require more than a reiteration of its value.

---

**Progressive Medicine,** quarterly, edited by H. A. Hare and L. F. Appleman, Philadelphia. Published by Lea & Febiger, Philadelphia, $6 per year. Vol. 20, No. 3, Sept. 1, 1917.

The present issue contains a review of Diseases of the Thorax by Wm. Ewart; Dermatology & Syphilis by Wm. S. Gottheil; Obstetrics by Edward P. Davis; Nervous System by Wm. G. Spiller.

**Diseases of the Spleen and Their Remedies Clinically Illustrated.** J. Compton Burnett, M. D., London, Am. edition published by Boericke & Tafel, Philadelphia, 83 pages, $1.

Along with a clinical review of diseases, the author discusses the appropriate homoeopathic remedies.

---

**War Shock,** The Psycho-neuroses of War, M. D. Eder, B. S., M. R. C. S., L. R. C. P., Late Temporary Captain, R. A. M. C. Published by P. Blakiston's Son & Co., Philadelphia, 164 pages, $1.75.

Of 100 cases, of neurotic nature, 30 gave a history of previous personal or family taint, 70 were distinctly due to war shock. The various manifestations of hysteria are especially well discussed.

---

**Ammunition for Final Drive on Booze.** Louis Albert Banks, M. D., Boston. Funk & Wagnalls Co., N. Y., 402 pages.

It is to be regretted that the title is itself somewhat intemperate and that the ammunition is rather oratoric than statistic. Statistics are not neglected entirely but a work of this kind, issued at present, ought to deal more seriously with the statistic arguments of various kinds promulgated by the liquor interests. For example, in spite of the recent triumphs of prohibition in the way of legislation, a large increase in the consumption of liquors for the year 1916, has been claimed. If this is a fact, the title is too optimistic. Indeed, we fear that we are very far from the final drive and that years of hand-to-hand combat are still necessary.

---

**Hand Book of Physiology,** W. D. Halliburton, M. D., LL.D., F. R. C. P., F. R. S., London. P. Blakiston's Son & Co., Philadelphia, 930 pages, $3.50. 13th edition.

This book is the lineal descendent of the physiology issued by Wm. Senhouse Kirkes of St. Bartholomy's Hospital, in 1848; and is, indeed, the 26th edition of that work. The original book was no mean contribution to medical science but had 705 pages and 97 illustrations. Various editors took charge of the revision from the 4th to the 13th edition, the next, in 1896 being the first issue under the present distinguished author. It is scarcely necessary to repeat the praise given to earlier editions as Halliburton's work has become a standard. What impresses us most forcibly is the fact that the advance of medical science does not necessarily imply an increase in volume or complexity. Indeed, it rather conduces to simplicity and brevity. A definitely established fact, whose relations to other facts are known, can be stated more briefly and comprehended more easily than a supposition, especially when it later is shown to involve fallacies.

---

**Malingering** or The Simulation of Disease, A. Bassett Jones and Llewellyn J. Llewellyn, with a chapter on Malingering in Relation to the Eye by W. M. Beaumont. P. Blakiston's Son & Co., Philadelphia. 708 pages, $7.

While this subject has been frequently treated in brief articles in medical and medico-legal periodicals and alluded to in various text books, we doubt whether a systematic work of comparable scope has hitherto appeared in English. The historic review carries us back into ancient literature and, under present war conditions, it is interesting to note that the feigning of disease to avoid military service was common among the Greeks, at first punishable by death, later by exposure to public gaze in female garments. The Romans cut off their thumbs instead of their trigger fingers. The influence of legislation, especially regarding various forms of insurance, is discussed at considerable length. While this book is especially timely on account of the large number of examinations being made with regard to fitness for military service, malingering is not so frequent to avoid military duty as for other reasons such as actions for damage or to secure insurance, for the sake of securing sympathy or attention or support, etc., nor is the view-point of the authors essentially military. The magnitude of the present work is not so much due to development of details as to the fact that malingering covers almost every disease and involves almost every system and apparatus of the body. One point should be especially emphasized: Everyone who, like the reviewer, is surprised that a volume of this size could be prepared on the subject, ought to read it. The authors have not produced so large a book simply by padding the comparatively brief notes on the subject which represent the knowledge of those who feel such a surprise. They are dealing with a subject which is very much larger than many otherwise well informed physicians imagine.

